# Association between watching eating shows and unhealthy food consumption in Korean adolescents

**DOI:** 10.1186/s12937-024-00961-1

**Published:** 2024-06-04

**Authors:** Min Jeong Joo, Dan Bi Kim, Jisu Ko, Jae Hyeok Lim, Eun-Cheol Park, Jaeyong Shin

**Affiliations:** 1 Department of Public Health, Graduate School, Yonsei University, Seoul, Republic of Korea; 2 Institute of Health Services Research, Yonsei University, Seoul, Republic of Korea; 3 Department of Preventive Medicine, Yonsei University College of Medicine, 50 Yonsei-to, Seodaemun-gu, Seoul, 03722 Republic of Korea

**Keywords:** Eating show, Mukbang, Cookbang, Unhealthy eating habits, South Korean adolescents, Fast food consumption, Sugar-sweetened beverages, High caffeinated beverages

## Abstract

**Background:**

Eating habits formed during adolescence greatly influence the maintenance of health in adulthood. With the recent development of social media and easy access to the Internet, adolescents watch plenty of food videos, particularly Mukbang and Cookbnag(eating show)content. This media genre’s impact on food choices has been covered in several studies; however, studies on unhealthy eating habits directly related to adolescents’ exposure to eating shows are insufficient.

**Methods:**

For this study, we used data from the 18^th^ Korea Youth Risk Behavior Survey conducted in 2022 and finalized 50,451 participants. The extent of exposure to eating show media over the course of a week, as well as the consumption of fast food, sugar-sweetened beverages (SSBs), and high caffeinated beverages within that week were measured through self-reporting questionnaires. We classified the participants into two groups based on their frequency of watching eating shows. A multiple logistic regression analysis was performed to investigate the association between eating show and unhealthy food consumption.

**Results:**

For both males and females, eating show exposure was strongly associated with the consumption of fast food (male: OR:1.37, 95% CI:1.26–1.49; female: OR:1.46, 95% CI:1.36–1.57), SSB (male: OR:1.42, 95% CI:1.26–1.60; female: OR:1.51, 95% CI:1.35–1.70), and high caffeinated beverage (male: OR:1.30, 95% CI:1.23–1.37; female: OR:1.24, 95% CI:1.18–1.31). It was observed that both sexes were more likely to frequently eat unhealthy food than students who did not watch eating shows.

**Conclusion:**

Among Korean adolescents, students exposed to eating shows, which primarily aim to entertain, were more likely to consume fast food, SSBs, and high caffeinated beverages. Therefore, this study's findings suggest that eating show could influence adolescents' food choices, highlighting the need for interest in emerging cultures and corresponding health policies.

**Supplementary Information:**

The online version contains supplementary material available at 10.1186/s12937-024-00961-1.

## Introduction

Nutritional habits formed during late childhood and adolescence are important as they continue into later stages of life [1]. Dietary habits formed during this period can affect adult physical development and cause age-related non-communicable diseases. However, adolescents whose eating habits have not yet been formed are more likely to be exposed to unhealthy foods than adults [2], as they are attracted to the positive factors inherent in instant food, such as convenience and the ability to enjoy it with friends [3]. The foods easily consumed by adolescents, such as fast food, sugar-sweetened beverages(SSB), and high caffeine beverages, are can led to various health issues, including obesity [4], hypertension [5], diabetes [6], depressive symptoms [7], cardiovascular diseases, and sleep disorders [8]. Therefore, there is a need for continued attention to adolescents' unhealthy food intake. There are various reasons why students choose these unhealthy foods, but among them, we have observed an association with media exposure.

According to previous research, regardless of content, digital media exposure influences adolescents' dietary intakes [9–13]. However, digital environments increasingly shape the surroundings of adolescents [14–16]; hence, understanding not only the form of media, but also the emergence and trends of various social media platforms that can influence adolescents' dietary habits is becoming increasingly important.

Various content available on the new media and whether it affects teenagers’ unhealthy food intake can help us understand their eating habits in the digital age. “Mukbang”, a Korean neologism, has gained prominence and immense popularity on video platforms. Mukbang is a type of online visual broadcast where creators or hosts consume various types of food while interacting with the audience on multiple online streaming platforms [17–19]. Another Korean neologism, “Cookbang,” meaning “cooking broadcast” or “broadcast of cooking and eating [20]” is also gaining popularity among adolescents. Video creators produce content that involves not only eating food or trying new food items, but also introducing various dishes and sharing recipes. Mukbangs and Cookbangs (eating shows) differ from cooking education programs or food advertisements in that their primary objective is to captivate viewers' interests. Eating shows showcase large quantities food consumption or displaying unhealthy food intake [21], potentially tempting viewers to overeat or adopt similar behavior [22]. This frequent exposure to unhealthy eating behaviors can distort negative eating behaviors as normal eating habits. Moreover, adolescents' unhealthy eating behaviors may have an impact on their health later in life and even during adulthood.

Recent research on the dietary habits of adolescents emphasizes the importance of healthy eating habits [23] and primarily investigates the relationship between habits such as the consumption of fast food, SSBs and high caffeinated beverages [24–27] and individual health. However, there is a lack of research on videos focused on consuming food for entertainment purposes and unhealthy food intake among adolescents. Therefore, this study examined the relationship between adolescents’ exposure to eating shows and their consumption of fast food, SSBs, and high caffeinated beverages.

## Methods

This is cross-sectional study that examines the relationship between the consumption of fast food, SSBs, and high-caffeine drinks and exposure to eating shows among Korean adolescents. This study was approved by the institutional review board of the Korea Disease Control and Prevention Agency (KDCA).

### Data

The data used in this study were obtained from the 18th Korea Youth Risk Behavior Survey (KYRBS) [28] conducted from August to October 2022 among Korean youth aged 12 to 18 in Korea. The survey was prepared with consideration for reliability and validity and reviewed by experts from the KDCA, and the Ministry of Education and the Ministry of Health and Welfare. The questionnaire comprised 114 questions on behaviors including smoking, drinking, and physical activity [29]. The survey was individually conducted using computers and mobile devices supervised by trained teachers and students provided their own answers. Students with long-term absences or who could not participate themselves, such as special education students or those with reading disabilities, were excluded. To maintain the representativeness of Korean youth, the survey drew samples from all regions of the country and school types in Korea with the supervision of the KDCA. A total of 400 middle schools and 400 high schools were selected. The sample schools were selected using permanent random sampling within each stratum. Then, one classroom per grade level was randomly chosen. All students in the chosen classrooms were surveyed [30].

### Study population

The study population comprised 56,213 students. The response rate of the surveyed students was 92.2%, resulting in the exclusion of 4,363 nonparticipants. Non-participation was primarily due to the additional workload on the teachers responsible for the survey and limited access to computers or mobile devices for evaluation. The final participant group initially included 51,850 students and was sampled to represent the youth population in South Korea. In our study, after removing data from 1,399 students with missing information, the final dataset comprised 50,451 students, covering 97.3% of Korean youth.

### Variables

In the current study, we investigated whether adolescents’ exposure to eating shows affects their unhealthy eating habits. The participants answered questions about how often they watched eating shows over the past 12 months. The response options were “I don’t watch at all”, “less than once a month”, “1 to 3 times a month”, “1 to 2 times a week”, “3 to 4 times a week”, “5 to 6 times a week” and “every day.” We classified them into the high (viewed more than once a week) and low group (viewed less than 3 times a month and none) based on the frequency of watching eating shows. Thereafter, for further analysis, the groups were divided into those who did not watch eating shows within seven continuous days, those who watched it less than twice a week, those who watched it three to six times a week, and those who watched it every day [31, 32].

Unhealthy food consumption was measured by assessing the frequency of fast food, SSBs, and high caffeinated beverage consumption. The participants were asked about their fast-food intake in the past seven days, and those who reported “once a week or more” were categorized as fast-food consumers. Similarly, SSBs (excluding zero sugar) and high caffeinated beverages consumption was determined based on the frequency of intake, with participants who responded “once a week or more” considered SSBs and high caffeinated beverages consumers.

The following independent variables were included in the analysis sociodemographic factors, age (7–9 grade: middle school, 10–12 grade: high school), region (urban, suburban, or rural), household economic level (high, middle, or low), school performance(high, middle, or low), and living type (with parents or others). Health-related factors included smoking status(yes or no), current alcohol use (yes or no), physical activity(none, 1– 3 days, 4– 6 days, or 7 days), stress level awareness(high, middle, or low), and body mass index(obesity, overweight, normal, or underweight by BMI levels). All variables were based on self-reported data.

### Statistical analysis

A descriptive analysis was conducted using the chi-square test to examine the distribution of general characteristics within the study population. After accounting for potential confounding variables, multiple logistic regression analyses were performed to explore the association between eating show exposure and the frequency of unhealthy food consumption among Korean adolescents. Owing to the differences in physical conditions between male and female students, all analyses were stratified by sex [33]. Subgroup analyses were performed to investigate the combined effects of each covariate on the risk of unhealthy food consumption and eating show exposure. Odds ratios (ORs) and 95% confidence intervals (CIs) were calculated to compare the data of participants who consumed unhealthy foods. The variables were clustered, stratified, and weighted to accommodate the small number of participants retained in the final analysis. All analyses were performed using SAS software (version 9.4; SAS Institute, Cary, NC, USA), and results were considered statistically significant *p*-values < 0.05.

## Results

Table 1 shows the general characteristics of the study population. Of the 50,451 participants, 25,747 were male students and 24,704 were female students. In the past seven days, 37.3% (*n* = 9,614) of male students and 48.2% (*n* = 11,897) of female students watched eating shows more than one time in a week. The consumption proportion of fast food, SSBs, and high caffeinated beverages by sex showed a similar distribution.

**Table 1:** Demographic characteristics of study participants

Category	Total	Male		Female	
	**N**	**N**	**%**	**N**	**%**
**Total**	**50,451**	25,747	51	24,704	49.0
**Eating show**					
Low (≤ 3 times/month or none)	28,940	**16,133**	62.7	12,807	51.8
High (≥ 1 times/week)	21,511	**9,614**	37.3	11,897	48.2
**Grade**					
7—9	27,308	**13,852**	53.8	13,456	54.5
10—12	23,143	**11,895**	46.2	11,248	45.5
**Region**					
Urban	21,669	**11,020**	42.8	10,649	43.1
Suburban	25,106	**12,764**	49.6	12,342	50.0
Rural	3,676	**1,963**	7.6	1,713	6.9
**Household economic level**					
High	21,345	**11,544**	44.8	9,801	39.7
Middle	23,573	**11,464**	44.5	12,109	49.0
Low	5,533	**2,739**	10.6	2,794	11.3
**School performance**					
High	19,626	**10,225**	39.7	9,401	38.1
Middle	15,159	**7,460**	29.0	7,699	31.2
Low	15,666	**8,062**	31.3	7,604	30.8
**Living type**					
with parents	47,986	**24,285**	94.3	23,701	95.9
Others	2,465	**1,462**	5.7	1,003	4.1
**Smoking**					
No	48,341	**24,263**	94.2	24,078	97.5
Yes	2,110	**1,484**	5.8	626	2.5
**Current alcohol use**					
No	44,055	**21,980**	85.4	22,075	89.4
Yes	6,396	**3,767**	14.6	2,629	10.6
**Physical activity**					
None	16,666	**6,485**	25.2	10,181	41.2
1—3 days	21,540	**10,631**	41.3	10,909	44.2
4—6 days	8,802	**6,010**	23.3	2,792	11.3
7 days	3,443	**2,621**	10.2	822	3.3
**Stress recognition level**					
High	20,696	**9,128**	35.5	11,568	46.8
Middle	21,189	**11,268**	43.8	9,921	40.2
Low	8,566	**5,351**	20.8	3,215	13.0
**BMI**^**a**^					
Obesity	6,332	**4,096**	15.9	2,236	9.1
Overweight	4,745	**2,779**	10.8	1,966	8.0
Normal	35,019	**16,945**	65.8	18,074	73.2
Underweight	4,355	**1,927**	7.5	2,428	9.8

ªBMI body mass index; underweight (BMI < 5th percentile), normal (5th < BMI ≤ 85th percentiles), overweight (85th < BMI ≤ 95thpercentiles), and obese (BMI > 95th percentile)

Table 2 presents the association between eating show exposure and unhealthy food consumption. For both male and female students, high eating show exposure was strongly associated with the consumption of fast food (male students: OR:1.37, 95% CI:1.23–1.49; female students: OR:1.46, 95% CI:1.36–1.57), SSBs (male students: OR:1.42, 95% CI:1.26–1.60; female students: OR:1.51, 95% CI:1.35–1.70), and high caffeinated beverages (male students: OR:1.30, 95% CI:1.23–1.37; female students: OR:1.24, 95% CI:1.18–1.31). Excluding fast food consumption among male students, those who do not live with their parents exhibited higher ORs of consuming unhealthy food within a week compared to students living with their parents. However, these differences were not statistically significant.

**Table 2:** Results of factors associated between eating show and unhealthy food consumption

Variablesª	Unhealthy food consumption													
	**Male**								**Female**					
	**Fast food**		**Sugar sweetened beverage**				**High caffeinated beverage**		**Fast food**		**Sugar sweetened beverage**		**High caffeinated beverage**	
	**OR**	**95% CI**	**OR**	**95% CI**			**OR**	**95% CI**	**OR**	**95% CI**	**OR**	**95% CI**	**OR**	**95% CI**
**Eating show**														
Low (≤ 3 times/month or none)	1.00		1.00				1.00		1.00		1.00		1.00	
High (≥ 1 times/week)	1.37	(1.26-1.49)	1.42	(1.26-1.60)			1.30	(1.23-1.37)	1.46	(1.36-1.57)	1.51	(1.35-1.70)	1.24	(1.18-1.31)
**Living type**														
With parents	1.00		1.00				1.00		1.00		1.00		1.00	
Others	0.85	(0.71-1.02)	1.23	(0.95-1.60)			1.00	(0.87-1.14)	1.05	(0.84-1.31)	1.26	(0.95-1.67)	1.08	(0.91-1.29)

ªAdjusted for Grade, Region, Household Economic Level, School Performance, Smoking, Current Alcohol Use, Physical Activity, Stress Recognition Level, BMI

Table 3 shows the relationship between exposure to eating shows more than once a week and unhealthy food consumption. Both male and female students in grades 7–9 were observed to be at a higher risk of consuming unhealthy food. Moreover, even for students who live with their parents, those excessively exposed to eating shows had a higher likelihood of consumption of fast food (male students: OR:1.39, 95% CI:1.27–1.51; female students: OR:1.46, 95% CI:1.36–1.58), SSBs (male students: OR:1.44, 95% CI:1.27–1.62; female students: OR:1.52, 95% CI:1.34–1.70), and high caffeinated beverages (male students: OR:1.30, 95% CI:1.22–1.37; female students: OR:1.25, 95% CI:1.19–1.32) compared to those who had low exposure.

**Table 3:** Results of subgroup analysis stratified by independent variables

**Variablesª**		**Eating show**					
		**Fast food**		**Sugar sweetened beverage**		**High caffeinated beverage**	
	**low**	**high**		**high**		**high**	
	**OR**	**OR**	**95% CI**	**OR**	**95% CI**	**OR**	**95% CI**
**Male**							
** Grade**							
7—9	1.00	1.53	(1.37–1.72)	1.77	(1.50–2.09)	1.34	(1.24–1.44)
10—12	1.00	1.21	(1.07–1.37)	1.14	(0.96–1.35)	1.26	(1.17–1.36)
** Living type**							
With parents	1.00	1.39	(1.27–1.51)	1.44	(1.27–1.62)	1.30	(1.22–1.37)
Others	1.00	1.11	(0.80–1.53	1.13	(0.68–1.89)	1.39	(1.12–1.72)
**Female**							
** Grade**							
7—9	1.00	1.55	(1.41–1.71)	1.55	(1.32–1.82)	1.32	(1.23–1.41)
10—12	1.00	1.36	(1.21–1.52)	1.46	(1.24–1.74)	1.18	(1.09–1.28)
** Living type**							
With parents	1.00	1.46	(1.36–1.58)	1.51	(1.34–1.70)	1.25	(1.19–1.32)
Others	1.00	1.41	(0.99–2.00)	1.68	(0.86–3.30)	1.05	(0.77–1.44)

ªAdjusted for Region, Household Economic Level, School Performance, Smoking, Current Alcohol Use, Physical Activity, Stress Recognition Level, BMI

**Fig. 1:**
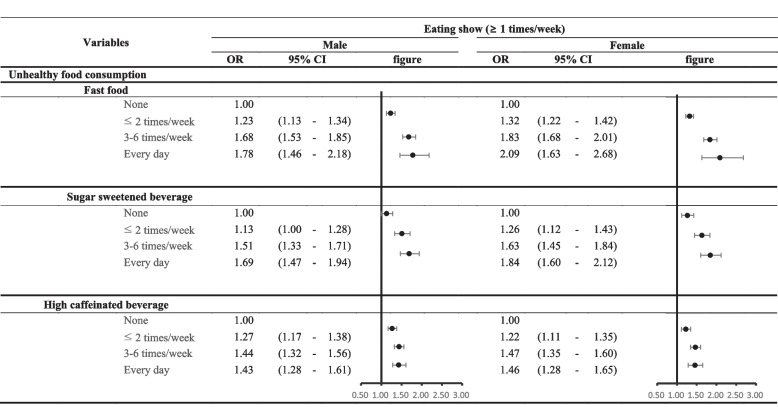
Association between eating show and each component of unhealthy food consumption

Figure 1 presents the subgroup analysis results stratified by unhealthy food consumption. It was noted that when both male and female students watched eating shows, the odds ratio for the frequency of fast food consumption increased (male students: OR: 1.78, 95% CI: 1.46–2.18; female students: OR: 2.09, 95% CI: 1.63–2.68). This trend was also consistent with SSB intake (male students: OR: 1.69, 95% CI: 1.47–1.94; female students: OR: 1.84, 95% CI: 1.60–2.12). However, those who watched eating shows were most likely to consume high caffeinated beverages three to six times per week compared to those who did not watch eating shows (male students: OR: 1.44, 95% CI: 1.28–1.61; female students: OR: 1.46, 95% CI: 1.28–1.65).

Table 4 shows the association between unhealthy food consumption and each component of eating show exposure. As the frequency of eating show exposure increased among both male and female students, the odds of eating unhealthy food increased compared to students who did not watch eating shows at all in the past seven days. However, male students’ consumption of fast food and SSBs, and female students’ consumption of SSBs showed the lowest odds ratio when watching eating shows every day.

**Table 4:** Association between unhealthy food consumption and each components of Eating show

Variables	Fast food		Sugar sweetened beverage		High caffeinated beverage	
**Male**	**OR**	**95% CI**	**OR**	**95% CI**	**OR**	**95% CI**
**Eating show**						
None	1.00		1.00		1.00	
1–2 times/week	1.45	(1.30 – 1.62)	1.63	(1.36 – 1.94)	1.23	(1.14 – 1.33)
3–6 times/week	1.46	(1.29 – 1.65)	1.48	(1.23 – 1.78)	1.36	(1.26 -).47
Every day	1.10	(0.93 – 1.29)	1.03	(0.84 – 1.27)	1.35	(1.21 – 1.51)
P for trend		< .0001		0.0006		< .0001
**Female**	**OR**	**95% CI**	**OR**	**95% CI**	**OR**	**95% CI**
**Eating show**						
None	1.00		1.00		1.00	
1–2 times/week	1.42	(1.29 – 1.57)	1.56	(1.33 – 1.82)	1.15	(1.07 – 1.23)
3–6 times/week	1.48	(1.34 – 1.64)	1.67	(1.43 – 1.96)	1.27	(1.18 – 1.36)
Every day	1.50	(1.32 – 1.72)	1.20	(0.99 – 1.45 )	1.42	(1.29 – 1.57)
P for trend		< .0001		< .0001		< .0001

## Discussion

The study found that adolescents who watched eating shows which focused on consuming food for entertainment purposes within a week after adjusting for potential covariates, had a higher risk of eating unhealthy food than those who did not.

Previous studies have emphasized the importance of environmental changes [34] and appropriate interventions by parents [35–37] and schools [38] to improve the dietary habits of adolescents, who are highly influenced by their surroundings. This education aimed to teach food literacy among adolescents and encourage them to read and understand food labels [39], cook healthy snacks [40], and adopt habits such as consuming vegetables and fruits [41], which are crucial for a healthy diet. However, our findings indicate that students surrounded by constant digital advancements require a different approach to overcoming unhealthy eating habits.

In our study, students living with their parents who watched eating shows were found to have a higher likelihood of having unhealthy food than those who did not watch eating shows. This finding is supported by previous studies which showed that even with parental involvement, those who watched food eating content on television [42], social networking apps, or other sites were more likely to consume the foods that the influencers were advertising [43] or the unhealthy foods that they saw [44, 45], than those who were not exposed. These findings suggest that the content of videos consumed by students, even those living with their parents, can influence their eating habits. Interestingly, when adolescents are exposed to and understand information about healthy foods such as vegetables or fruits, and perceive them favorably, they respond positively to healthy products [46, 47]. These results underscore the significance of the content adolescents consume, suggesting directions to promote healthy eating habits among them.

In both male and female students in grades 7–9, those who watched an eating show more than once a week had a higher risk of eating unhealthy food compared to those who rarely watched an eating show. In previous studies, it was found that students in lower grades are influenced by their friends' food consumption and are also influenced by their friends' opinions on their own food consumption [48]. Furthermore, although cognitive abilities regarding food are crucial factors in food choices [49], during this period, food literacy tends to be lower compared to other times [50]. This suggests that the influence of eating shows may depend on students' educational levels and socialization.

Interestingly, despite female students generally being more sensitive about their body image and paying more attention to others' perceptions [51], female students who watched eating shows did not exhibit a different risk of consuming unhealthy food compared to male students. This is consistent with previous research examining media exposure and food consumption by sex [52, 53], indicating that both female and male students can be influenced in their dietary habits by eating show exposure. Therefore, there is a continued need for ongoing attention to the exposure of adolescents to eating show. For students who are in the process of developing healthy eating habits, interventions such as inserting warning messages before the start of eating show or ensuring accurate dissemination of information about the food being consumed are required, along with policy interest in addressing these concerns [54, 55].

This study’s findings underscore the importance of investigating adolescent exposure to eating shows and unhealthy food consumption. However, it is essential to acknowledge several study limitations. First, this study relied on a cross-sectional design, which means that it could only identify associations between variables and could not establish causal relationships. Further research employing longitudinal or experimental designs is thus necessary to confirm causality. Second, exposure to eating shows and unhealthy food consumption were assessed through self-reports. Therefore, there were limitations in measuring the types and content of eating show content participants watched, as well as their exact screen times. Additionally, we were unable to measure participants' exact intake amounts of fast food, SSBs, and high caffeinated beverages. Depending on the participants’ recollection and willingness to report accurately, response biases and inaccuracies may have influenced the results. Future studies should thus incorporate more objective measures or complementary data sources to enhance the validity of the assessments.

Despite these limitations, this study had several strengths. First, it employed a well-structured analysis stratifying exposure to eating shows and unhealthy food consumption. This approach enhanced the quality of the data and makes it valuable for future research in this area. Second, the study benefited from a representative sample, which means that its findings offer insights into the broader situation of South Korean adolescents. These findings can be instrumental in shaping more effective public health policies and interventions to address issues related to media influence on adolescent eating habits.

## Conclusion

This study examined the relationship between eating show as new media content of and unhealthy food consumption among adolescents. Students who watched eating shows more than once a week had a higher risk of eating unhealthy foods than those who did not watch them. These findings suggest that exposure to eating show, which primarily aim to entertain, can influence adolescents' food choices. Further research is needed to reveal the fundamental mechanisms behind the connection between eating habits by accurately measuring the food consumption and screen time adolescents watching food entertainment content.

### Supplementary Information

Supplementary Material 1.

Supplementary Material 2.

## Data Availability

The datasets generated during and /or analyzed during the current study are available in the 18th Korea Youth Risk Behavior Survey (KYRBS) 2022 (https://www.kdca.go.kr/yhs/).

## Abbreviations

SSB: Sugar-sweetened beverage
KYRBS: Korea Youth Risk Behavior Survey
KCDC: Korea Centers for Disease Control and Prevention
OR: Odds ratio
CI: Confidence interval
